# VeriAbs: A Tool for Scalable Verification by Abstraction (Competition Contribution)

**DOI:** 10.1007/978-3-030-72013-1_32

**Published:** 2021-02-26

**Authors:** Priyanka Darke, Sakshi Agrawal, R. Venkatesh

**Affiliations:** 8 Eindhoven University of Technology, Eindhoven, The Netherlands; 9 Aalborg University, Aalborg East, Denmark; TCS Research, Pune, India

## Abstract

VeriAbs is a strategy selection-based reachability verifier for C programs. The selection of a suitable strategy is from a pre-defined set of strategies and by taking into account the syntax and semantics of the code to be verified. This year we present VeriAbs version 1.4.1 in which a novel preprocessor to strategy selection is introduced. The preprocessor checks for the feasibility of performing a lightweight slicing of the input code using function call graph and variable reference information. By this if the program is found to be *sliceable*, sub-programs or slices are generated, and the known strategy selection algorithm of VeriAbs is applied to each slice. The verification results of each slice are then composed to derive that of the entire program. This compositional verification has improved the scalability of VeriAbs and presented in this paper.

## 1 Verification Approach

VeriAbs is a C program verifier using a portfolio of twelve verification techniques [2]. These techniques are organized into four strategies as shown in Figure 1. Each of the strategies is defined such that it benefits verification of a specific type of programs. A program type is identified by a strategy selector based on the following code-structural and variable-data properties: (1) unstructured control flow, (2) loops with arrays, (3) short input ranges, and (4) numerical loops in code. The strategy selector looks for these properties in the given order and assigns a verification strategy to the code. For this it uses code-structure and interval analyses [2]. If the assigned strategy is unable to verify the program, it exits unless if the program contains arrays. In that case it selects the *default* strategy corresponding to numerical loops. Kindly refer to [2,3] for details on each verification technique implemented in VeriAbs.

The colored blocks in Figure 1 indicate the enhancements to the tool made this year and are explained next. The colored block with a dashed outline indicates that the component has been added for the first time in VeriAbs, and that with a solid outline indicates that a block that existed in older versions has been modified. The dashed arrows indicate information flow added this year. This information is the verification result of the respective strategy passed back to the slicer-analyzer explained in the next section. Besides these, there are changes in witness generation strategies and explained in the next section.

⋆ Jury member

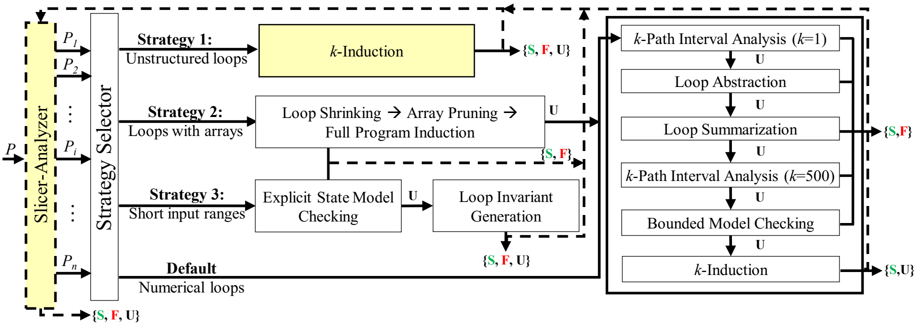

**Fig. 1.** VeriAbs Architecture (**S**: Program Safe, **F**: Property Fails, **U**: Unknown)

## 1.1 Tool Enhancements

*Slicer-Analyzer.* It has the following responsibilities: (1) checking the *sliceability* of input program $P$, (2) generating slices $P_1, P_2, \ldots, P_r$ if $P$ is sliceable, and (3) computing the verification result $R$ of $P$. Accordingly, the slicer-analyzer comprises of three parts. The first part checks for *sliceability*. Let *main* be the entry function of $P$. We define $P$ to be sliceable with respect to *main* if all distinct functions $f_1, f_2, \ldots, f_r$ directly called from *main* are defined in $P$, and are independent of each other. We define the functions called from *main* independent iff *main* is non-recursive; contains no loops or unstructured control flow [2]; there is no transitive dependence (made up control and data dependence) between calls to $f_1, \ldots, f_r$ in *main*; no two functions in $f_1, f_2, \ldots, f_r$ transitively call the same function; and if $F(f_i)$ is the union of $f_i$ and functions transitively called by $f_i$, then no two sets in $F(f_1), F(f_2), \ldots, F(f_r)$ refer to the same global variable in the program. That is, if $V(F(f_i))$ is the set of global variables referred by functions in $F(f_i)$ then $\forall m,n \mid 1 \le m \le r,\ 1 \le n \le r,\ m \ne n \Longrightarrow V(F(f_m)) \cap V(F(f_n)) = \emptyset$. The call graph and referred variables information is computed using call-trees, and a light-weight flow insensitive pointer analysis.

```
void main() {
 b=30,c=10;
 if(!a) f1();
 else if(b) f2();
 ...
}
f1(){c++;}
f2(){b=0;assert(b);}
```

**Fig. 2.** Input Code

If above stated conditions are satisfied then using concepts presented in [10], the body of *main* is sliced with respect to call(s) to $f_i$ to create the entry function $main^i$ of the executable slice $P_i$. Since *main* is sliced with respect to calls to $f_i$, $P_i$ will only have functions in $F(f_i)$ and $main^i$. That is, the set of functions in slice $P_i$ is given by $F(f_i) \cup main^i$. This way the set of all slices are generated by the second part of the slicer-analyzer. The proposed technique of slicing has the potential to greatly reduce the state space of the input program. This hypothesis is supported by experimental results presented later. The proposed slicing function uses control- and data-flow information local to *main*, hence it is lightweight.

Consider the example in Figure 2. One slice from this code is given in Figure 3. As seen, function *main* has been sliced with respect to the call to $f2$ in Figure 3

which contains the error. Function $f1$ need not be analyzed to find the error. This type of slicing is helpful in analyzing large code in which the verifier may run out of resources while analyzing an irrelevant function like $f1$.

```
void main() {
 b=30,c=10;
 if(!a) ;
 else if(b) f2();
 ...
}
f2(){b=0;assert(b);}
```

**Fig. 3.** One Slice

Next, VeriAbs applies its strategy selection to each slice $P_i, \forall i, 1 \leq i \leq r$ sequentially. The results of each slice are composed to compute $R$, the verification result of $P$, by the third part of the slicer-analyzer as follows: if an error trace is realized for any slice then $R$ is set to *failure*; if all slices are proved to be safe, then $R$ is set to *safe*; otherwise if none of the slices are found to be erroneous and there exists a slice that could not be verified, then $R$ is set to *unknown*.

This idea of slicing based on function call and variable reference information has been proposed for the first time. It is similar to a concept of clustering presented in [12]. Both these techniques partition a given application into independently executable slices. But [12] forms clusters with respect to un-called functions in the code base. The proposed sliceability criterion on the other hand focuses only on functions called from a given (entry) function *main*. It uses control- and data-flow analyses local only to the given function to slice it with respect to calls in its body. This in turn removes all functions not called from *main*. Another technique generates multiple backward slices at every calling context with respect to a property to be verified [8]. The proposed slicing technique however produces slices with respect to functions defined in $P$ and called from *main*.

*Witness Generation From Slices:* VeriAbs stores slices in the form of separate C programs. To generate a valid witness from a slice it is critical to report the correct line numbers in the witness [5]. The slicer-analyzer maintains correct line numbers in the slice with respect to the original code by adding #line directives to it. The directives are added at every point in the slice which reads values from the environment, starts a block of code, or contains a branching condition. The witness generated from such a slice in VeriAbs is valid with respect to the original program.

*Experimental results:* The proposed slicing led to VeriAbs successfully analyzing 120 additional programs in ReachSafety in SV-COMP'21. On the other hand it runs out of time while verifying eighteen programs that it could successfully analyze earlier. This is due to the additional time required to slice. Overall these values demonstrate the feasibility of this approach.

Next we present modifications made to existing components of VeriAbs.

*Strategy 1: Unstructured Control Flow.* The first strategy meant for programs with unstructured control flow, thus far executed two verification techniques in parallel. The two techniques were evolutionary test generation algorithms using grey box fuzzing [13], and $k$-induction with continuously refined invariants [6]. This year we do not use the first algorithm in strategy 1. The reason being that the time taken by it to generate useful error traces is very large. We observe that as the program complexity increases with the number of constraints, branching conditions, and/or non-determinism, so does the time to reach the error by the test evolution algorithm. This leads to the effect of no apparent advantage of the algorithm when applied in parallel with $k$-induction. We present our experimental observations of the given algorithm in [2]. On the other hand, not using this algorithm led to time savings and verification of a few additional programs. We continue to use this algorithm for non-reactive loops and for programs with inputs of short ranges (strategy 3) [2]. Here we allocate it an independent thread with no time limits, while results are obtained quickly for non-reactive loops.

*Witness Generation.* This year VeriAbs uses the same strategies as last year to generate violation witnesses [3]. For correctness witnesses VeriAbs derives invariants from the over-approximation techniques in its portfolio. To save time this year VeriAbs does not extract invariants from $k$-induction [6] and interpolation [11] to generate correctness witnesses. From amongst the impacted witnesses, this led to 12 fewer witnesses being validated than last year.

## 2 Software Architecture

VeriAbs uses Vajra to perform full program induction [7], American Fuzzy Lop [13] to perform test evolution with fuzzing, and CPAchecker v1.8 [6] in the first strategy for $k$-induction. For bounded model checking VeriAbs uses the C Bounded Model Checker (CBMC) v5.10 [9] with the Glucose Syrup SAT solver v4.0 [4]. All remaining program analyses are implemented in the TCS Research group's program analysis framework called Prism [12]. The slicer-analyzer and the strategy selector are partly implemented in perl.

## 3 Strengths and Weaknesses

The main strengths of VeriAbs lie in its (1) portfolio of sound verification techniques, and its ability to (2) perform a lightweight slicing, (3) classify programs based on structural and variable data properties of code, and (4) match these code properties with suitable verification techniques. The main weakness of VeriAbs lies in its lack of an integrated implementation of witness generation that can utilize invariants derived across all strategies or techniques. This is because the invariants are to be derived from various abstractions, some of which are generated by off-the-shelf tools, and not yet extracted.

## 4 Tool Setup and Configuration

The VeriAbs SV-COMP 2021 executable is available for download at https://gitlab.com/sosy-lab/sv-comp/archives-2021/-/tree/master/2021/veriabs.zip. To install the tool, download the archive, extract its contents, and then follow the installation instructions in `VeriAbs/INSTALL.txt`. To execute VeriAbs, the user needs to specify the property file using the `--property-file` option. The witness is generated in the current working directory as `witness.graphml`. VeriAbs participated in the ReachSafety category of SV-COMP 2021. The BenchExec wrapper script for the tool is `veriabs.py` and the benchmark description file is `veriabs.xml`. A sample command is as follows:

```
VeriAbs/scripts/veriabs --property-file reach-safety.prp a.c
```

## 5 Software Project and Contributors

Few members of the Foundations of Computing group at TCS Research [1] maintain VeriAbs. They can be contacted at veriabs.tool@tcs.com. We thank past developers of VeriAbs, creators of Prism [12], Vajra, CPAchecker and CBMC. We specially thank Bharti Chimdyalwar, Shrawan Kumar and Ulka Shrotri for their insightful reviews.
